# Understanding COVID-19-associated endothelial dysfunction: role of PIEZO1 as a potential therapeutic target

**DOI:** 10.3389/fimmu.2024.1281263

**Published:** 2024-02-29

**Authors:** Xiaoting Zhang, Jinhai Liu, Xiaoming Deng, Lulong Bo

**Affiliations:** Faculty of Anesthesiology, Changhai Hospital, Naval Medical University, Shanghai, China

**Keywords:** COVID-19, SARS-CoV-2, endothelial dysfunction, immunothrombosis, PIEZO1, therapeutic target.

## Abstract

Coronavirus disease 2019 (COVID-19) is an infectious disease caused by severe acute respiratory syndrome coronavirus 2 (SARS-CoV-2) infection. Due to its high infectivity, the pandemic has rapidly spread and become a global health crisis. Emerging evidence indicates that endothelial dysfunction may play a central role in the multiorgan injuries associated with COVID-19. Therefore, there is an urgent need to discover and validate novel therapeutic strategies targeting endothelial cells. PIEZO1, a mechanosensitive (MS) ion channel highly expressed in the blood vessels of various tissues, has garnered increasing attention for its potential involvement in the regulation of inflammation, thrombosis, and endothelial integrity. This review aims to provide a novel perspective on the potential role of PIEZO1 as a promising target for mitigating COVID-19-associated endothelial dysfunction.

## 1 Introduction

Coronavirus disease 2019 (COVID-19) has become a major global health concern caused by the severe acute respiratory syndrome coronavirus 2 (SARS-CoV-2), which primarily spreads through respiratory droplets (1). With the emergence of highly transmissible variants, the COVID-19 pandemic has caused a significant healthcare burden worldwide, affecting millions of people. SARS-CoV-2 infection can lead to various clinical manifestations, ranging from asymptomatic cases to life-threatening illnesses. In addition to respiratory symptoms, a variety of extrapulmonary manifestations have also been reported (2). Recent studies suggest that endothelial dysfunction and immunothrombosis are key pathogenic mechanisms in COVID-19 (3, 4). However, effective therapeutic management of COVID-19 patients remains challenging. Understanding the pathogenesis and pathophysiological mechanisms underlying COVID-19-associated endothelial dysfunction is crucial for identifying potential therapeutic targets.

Piezo1, a member of the Piezo ion channel family, is expressed in vascular endothelial cells where it senses mechanical stimuli induced by blood flow and blood pressure. Over the past decade, Piezo1 has been shown to function as both a physiological regulator and a pathogenic factor in endothelial cells by modulating endothelial functions and barrier integrity (5–9). Interventions targeting Piezo1 in endothelial cells have demonstrated its role in various diseases, including ventilator-induced lung injury, hypertension, atherosclerosis, and thrombosis. Human PIEZO1 and PIEZO2 genes are located in the 16q24.3 region of chromosome 16 and the 18p11.22-p11.21 region of chromosome 18, respectively (10). Notably, Sukumar et al. (11) found that single nucleotide polymorphisms (SNPs) within the PIEZO1 gene are more prevalent in SARS-CoV-2 infected individuals. This suggests that PIEZO1 merits further study as a potential therapeutic target for COVID-19. In this review, we discuss how SARS-CoV-2 infection damages the endothelium and propose that PIEZO1 dysregulation could contribute to COVID-19-associated endothelial dysfunction, highlighting it as a promising therapeutic candidate.

## 2 COVID-19-associated endothelial dysfunctions

The ongoing battle against SARS-CoV-2 necessitates a deeper understanding of the intricate interplay between the virus and the host immune system. Following infection, the host immune response is activated, leading to the release of inflammatory signaling molecules and the recruitment of T lymphocytes, monocytes, and neutrophils (12, 13). This sustained immune response, coupled with epithelial injury, disruption of the endothelial barrier, and activation of coagulation, collectively contribute to diverse forms of tissue damage (14–16). Notably, clinicians are increasingly concerned about the emergence of cardiovascular complications in COVID-19 patients (17–20). Mounting evidence indicates that hemodynamic dysfunction, endothelitis, vascular leakage, and thrombosis play pivotal roles in the multi-organ damage observed in organs such as the lungs, heart, kidneys, and intestines (21). Consequently, resolving COVID-19-associated endothelial dysfunction has become an urgent priority.

### 2.1 COVID-19-associated hemodynamics dysfunction

Angiotensin-converting enzyme 2 (ACE2) is a surface membrane protein that is expressed in multiple tissues (22). It is widely known as an important enzyme in the cardiovascular system, which converts angiotensin II (Ang II) into angiotensin (1−7), and negatively regulates the renin-angiotensin system. ACE2 and angiotensin (1−7) are essential for endothelial cell function as they inhibit the inflammatory response (23, 24). Angiotensin (1−7) also participates in the activation of endothelial nitric oxide synthase (eNOS), which is mainly responsible for nitric oxide (NO) production and plays an important role in vasodilation (25, 26). Therefore, loss of ACE2 can lead to NO imbalance and vascular dysfunction.

During COVID-19, ACE2 is the main entry receptor for SARS-CoV-2 (27, 28). The active site domains of ACE2 are exposed on the extracellular surface, and SARS-CoV-2 can thus bind through the spike (S) protein on its surface, following the lock and key mechanism. Upon binding, transmembrane protease serine 2 (TMPRSS2), a cellular protease, cleaves and primes the S protein to further mediate viral uptake (29, 30). The virus then starts self-replicating. However, high-affinity SARS-CoV-2 competes with Ang II to bind the ACE2 receptor, leading to the accumulation of Ang II and causing an imbalance in the Ang (1−7)/Ang II ratio (31).

The accumulation of Ang II, accompanied by SARS-CoV-2 infection, can activate a transmembrane metalloproteinase called ADAM17, which mediates the ectodomain shedding of ACE2 (32, 33). Elevated soluble ACE2 (sACE2) levels can be observed in the blood circulation of patients with COVID-19 (34). ADAM17 cleaves the extracellular juxtamembrane region of ACE2 and releases its catalytically active extracellular domain of ACE2 into the extracellular environment, which may ultimately facilitate viral entry (35, 36). Owing to the ubiquitous expression of ACE2, TMPRSS2, and ADAM17 in various tissues, SARS-CoV-2 infection leads to multi-organ injuries (37). Moreover, the accumulating Ang II binds to the angiotensin type 1 receptor (AT1 receptor) and induces the internalization and downregulation of ACE2 (38). With the replication of SARS-CoV-2, the expression of ACE2 on the cell surface is downregulated, which reduces its vasodilatory effect in the vascular system. Prolonged vasoconstriction results in increased endothelial dysfunction and inflammation, which leads to severe cardiovascular injury. In a recent study, it was found that a significant proportion of patients hospitalized due to COVID-19 exhibited the production of autoantibodies against Ang II. The presence of these autoantibodies was strongly correlated with poorer blood oxygenation, dysregulated blood pressure, and overall increased severity of the disease (39). Furthermore, some COVID-19 patients also developed autoantibodies against AT1 and ACE2, leading to heightened proinflammatory responses and greater disease severity (40, 41). These findings provide valuable insights into the quantification of autoantibodies targeting crucial molecules in the renin-angiotensin pathway, *i.e.*, Ang II, AT1, and ACE2. Moreover, they highlight the significance of disturbances in vascular tone among COVID-19 patients.

### 2.2 COVID-19-associated endotheliitis and endothelial barrier breakdown

Endotheliitis and disruption of the endothelial barrier are crucial pathological mechanisms in COVID-19, contributing to increased vascular permeability and inflammation, which ultimately lead to multi-organ dysfunction (42). Electron microscopy observations have revealed that SARS-CoV-2 can directly infect endothelial cells (ECs), thereby potentially altering vascular homeostasis (21). In some COVID-19 patients, elevated levels of circulating ECs and soluble circulating endothelial derangement parameters have been detected, indicating EC apoptosis and breakdown of the endothelial barrier (43).

In severe cases of COVID-19, excessive production of pro-inflammatory cytokines, often referred to as a “cytokine storm,” contributes to the transition of the endothelial cell phenotype from a protective state to an inflammatory state. This transition is associated with increased vascular leakage, tissue damage, and immunothrombosis. As mentioned previously, hyperactivity of Ang II serves as a trigger for vasoconstriction and inflammation. Additionally, SARS-CoV-2 activates the NLRP3 inflammasome in monocytes, leading to the release of pro-inflammatory cytokines that further amplify endotheliitis (44). In severe cases, a delayed or impaired interferon type I response results in persistent viral load in the blood and exacerbates the inflammatory response (45).

SARS-CoV-2 infection can cause severe damage to the endothelial barrier. Activation of the NLRP3 inflammasome by SARS-CoV-2 increases the release of interleukin-1 beta (IL-1β), which suppresses cAMP formation and CREB-mediated transcription of VE-cadherin in ECs, consequently contributing to vascular leakage (46). Recent studies have shown that lactate induces vascular hyperpermeability by promoting cleavage and endocytosis of VE-cadherin through signaling via the lactate receptor GPR81 in endothelial cells (47). Lactate, as a major byproduct of glycolysis, has also been found to be elevated in severe COVID-19 patients (48, 49), indicating the involvement of metabolic changes in endothelial dysfunction. Furthermore, recruited neutrophils release excessive reactive oxygen species (ROS), which are exacerbated by reduced antioxidants due to viral infection, further intensifying endothelial injury (50).

### 2.3 COVID-19-associated immunothrombosis

Under homeostatic conditions, the glycocalyx and anticoagulants produced by ECs play a crucial role in preventing microvascular thrombosis and maintaining normal blood flow (51). However, SARS-CoV-2 infection disrupts vascular integrity, exposing thrombogenic basement membranes (52). Consequently, the activation of the coagulation cascade contributes to the development of immunothrombosis (53, 54).

Vascular damage and the inflammatory environment activate the tissue factor (TF) and extrinsic pathways. SARS-CoV-2 also triggers complement activation and elevates plasma TF levels (55–57). Neutrophil extracellular traps (NETs) are recognized as important mediators of tissue damage. The concentration of NETs is increased in samples from COVID-19 patients, including plasma, tracheal aspirate, and lung autopsy tissues. Neutrophils from COVID-19 patients release excessive NETs *in vitro*, and plasma from these patients triggers NET formation (58, 59). The enhanced release of NETs can activate the intrinsic coagulation pathway via factor XII. Neutrophil elastase and myeloperoxidase present in NETs cleave and inactivate natural anticoagulants, such as tissue factor pathway inhibitor and thrombomodulin (60, 61). Moreover, NETs promote platelet adhesion and activation (62). Complement cascade activation induces NETosis and platelet activation (63). Consequently, activated platelets, distorted neutrophils, aggregated NETs, and fibrin strands contribute to vascular occlusions. In response, fibrinolysis is activated, resulting in elevated levels of fibrin breakdown products.

**Figure 1:**
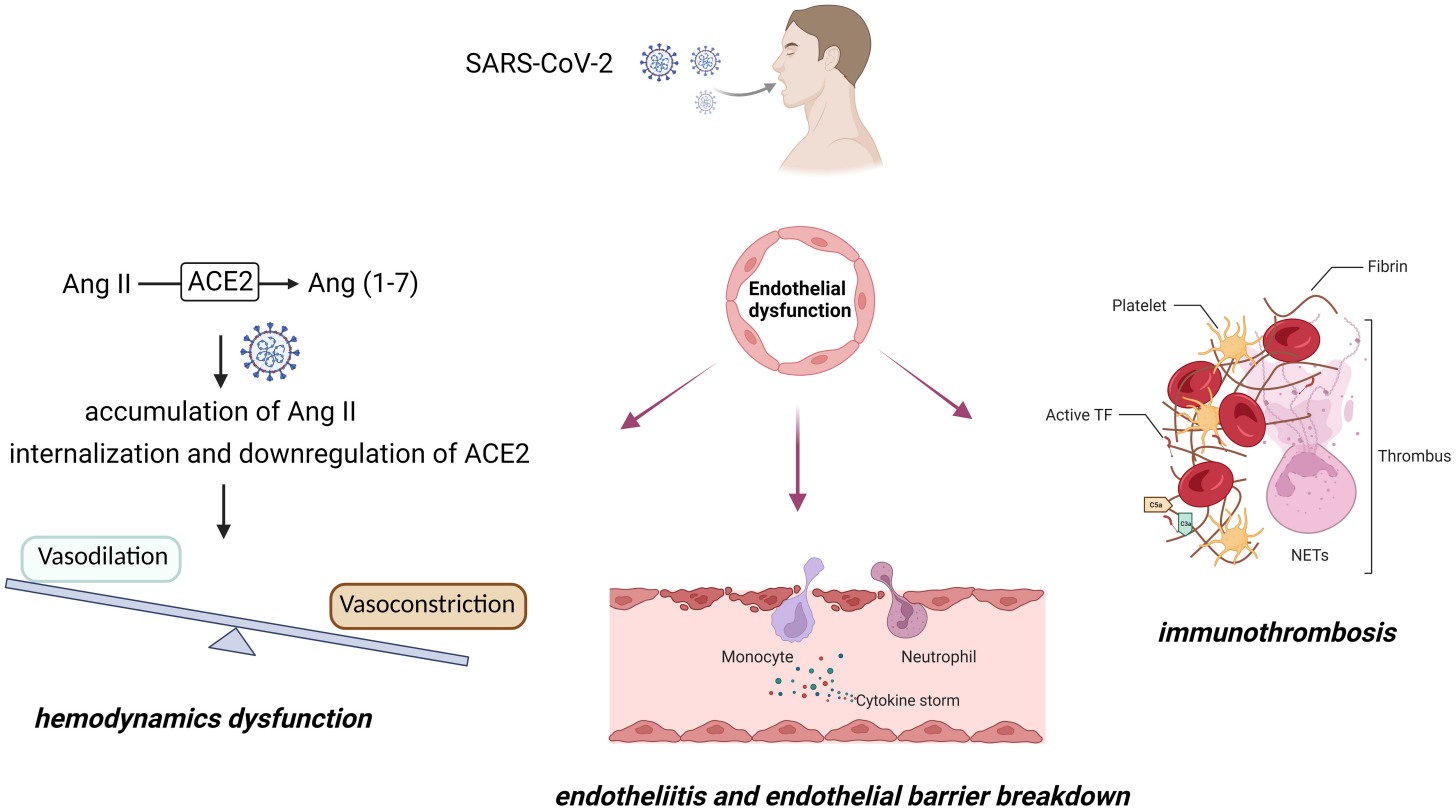
COVID-19-associated endothelial dysfunction.

Endothelial dysfunction is a prominent feature during the progression of COVID-19. This dysfunction leads to vasoconstriction, breakdown of the endothelial barrier, and a procoagulant state (**Figure 1**). Systemic impairment of microcirculatory function poses a significant threat to COVID-19 patients. Therefore, it is crucial to develop strategies that target endothelial cells, especially for patients with pre-existing endothelial conditions (*e.g.*, smoking, hypertension, and diabetes), who are at a higher risk of mortality.

(1) ACE2, the primary receptor for SARS-CoV-2, is a critical enzyme in the cardiovascular system responsible for the conversion of Ang II into angiotensin (1−7). In the context of COVID-19 infection, internalization and downregulation of ACE2 result in the accumulation of Ang II, thereby potentially contributing to the mediation of vasoconstriction and hemodynamic dysfunction. (2) SARS-CoV-2 infection triggers an immune response, characterized by the secretion of cytokines from recruited monocytes and neutrophils, subsequently leading to the induction of endotheliitis. The escalated oxidative stress, cytokine storm, and leukocyte adhesion to the endothelium contribute to severe endothelial barrier disruption. (3) Vascular damage and inflammatory states initiate the activation of tissue factor (TF) and the extrinsic coagulation pathway. The amplified release of neutrophil extracellular traps (NETs) can cleave and inactivate endogenous anticoagulants. Moreover, NETs play a pivotal role in facilitating platelet adhesion and activation. The aggregation of NETs, fibrin strands, deformed neutrophils, and activated platelets synergistically contribute to the genesis of vascular occlusions.

## 3 Mechanosensitive Piezo ion channels

Piezos, encompassing Piezo1 and Piezo2, are expressed in various tissues. Both channels are expressed on the cellular membrane and are encoded by the Piezo1 gene (family with sequence similarity 38A, Fam38A) and the Piezo2 gene (family with sequence similarity 38B, Fam38B), respectively (64). Mechanosensitive Piezo ion channels were initially identified as fundamental components of distinct mechanically activated cation channels in 2010 (65). The groundbreaking discoveries made by Ardem Patapoutian’s research group marked a significant milestone in this field, unveiling a realm of limitless possibilities. Subsequent extensive investigations have revealed an expanding repertoire of functions that extend beyond the realm of mechanotransduction processes. In recognition of his pioneering work in identifying these force receptors, Ardem Patapoutian was awarded the Nobel Prize in Physiology or Medicine in 2021.

### 3.1 Structure of Piezo1 ion channel

The core architecture of mouse Piezo1 consists of a homotrimeric complex exhibiting a propeller-like shape with a central cap, three peripheral blade-like structures, and three beams connecting the blades to the cap (66, 67). Additionally, the high-resolution three-dimensional cryo-electron microscopy revealed that each subunit of the Piezo1 homotrimeric complex encompasses 38 transmembrane (TM) segments, collectively summing up to 114 TM segments. In 2022, the mechanism of Piezo1 structural deformation in response to mechanical force within lipid bilayers was elucidated (68). The TM regions observed within the three-bladed structure of Piezo channels are unusually curved and shape a distinctive nano-bowl configuration. The researchers postulated a hypothesis that the application of force on the cell membrane may induce a flattening of the paddles, consequently resulting in an expansion of the membrane area. The findings presented in this study provide a comprehensive understanding of the intricate mechanistic pathway through which Piezo1 converts physical mechanical stimuli into bioelectric signals, consequently offering valuable insights into the mechanotransduction process.

### 3.2 Expression and functions of Piezo1 ion channel

Piezo1 represents a pivotal mechanically-activated cation channel that senses mechanical stimuli and plays critical roles in diverse physiological functions. Mechanically activated Piezo1 converts the mechanical stretch experienced by cardiomyocytes into intracellular Ca^2+^ and reactive oxygen species (ROS) signaling, thereby playing a key role in heart mechano-chemo transduction and the maintenance of normal cardiac function (69). In enterochromaffin cells, Piezo1 detects single-stranded RNA derived from the intestinal microbiota and facilitates serotonin production (70), which is relevant to bone and gut disorders. Moreover, Piezo1 is essential for vascular development, lymphatic development, red blood cell volume regulation, blood pressure control, arterial remodeling, iron metabolism, bone formation, and tissue inflammation (5, 71–75). Consequently, Piezo1 emerges as a potential therapeutic target for numerous diseases.

### 3.3 Unique features of Piezo1 compared to other MS channels

Several MS channels have been identified, including PIEZO, transient receptor potential (TRP) channels, epithelial sodium channels (ENaC), K_2P_ channels, and others (76). Each of these channels has unique structures and mechanotransduction mechanisms that align with their specific biological functions. In comparison to other MS ion channels, Piezo1 exhibits unique properties and activation characteristics. As described above, Piezo1 exhibits activation in response to various mechanical stimuli, including external poking, stretching, shear stress, local membrane tension, and intracellular forces mediated by myosin-II traction (68). Transient receptor potential vanilloid 4 (TRPV4) can be activated by osmotic changes, membrane stretch, chemical ligands, and temperature changes (77–79). K_2P_ channels respond to a range of stimuli, including protons, heat, stretch, diverse lipids, and pharmacological substances such as general anesthetics (80, 81). Among these MS channels, Piezo protein family has recently been established as the first bona fide mechano-gated cation channels in mammals, a groundbreaking discovery that has long been sought after (64). Piezo1 is characterized by its low-threshold, small-conductance, fast inactivation, and depolarization upon mechanical activation. It acts as a non-selective cationic channel that allows the permeation of sodium, potassium, and calcium ions (68, 82, 83). The activation of Piezo1 is notable for its exceptional mechanosensitivity, with a pressure of approximately -30 mmHg required for half-maximal activation and a lateral membrane tension of around 1.4 mN/m (64, 84). These values highlight the sensitivity of Piezo1 to mechanical stimuli. The unitary conductance, which measures the ability of a single opened channel to allow ion movement, is estimated to be about 20 pS for Piezo1 and 30 pS for Piezo2 (68, 85). As a mechanosensitive cation channel, Piezo1 is mainly permeable to calcium ions. Calcium plays a crucial role as a second messenger in numerous endothelial cell activities. Changes in intracellular calcium ion levels and ensuing signaling cascades are responsible for these processes. Under normal conditions, quiescent endothelial cells maintain low intracellular cytosolic free calcium concentrations. However, the initiation of calcium signaling can be induced by proinflammatory mediators, and elevated intracellular calcium concentrations can trigger diverse signaling pathways in both physiological and pathological conditions (68, 86). Upon opening, Piezo1 channels facilitate the entry of calcium ions, subsequently initiating downstream signaling pathways that are crucial for endothelial function and vascular homeostasis. Furthermore, studies have revealed that purified Piezo1 exhibits activation in an asymmetric bilayer but not in a symmetric bilayer, indicating its intrinsic sensitivity to membrane curvature (87). The distinctions in conductance and mechanical activation thresholds between Piezo1 and other mechanosensitive channels emphasize their unique contributions to physiological function.

### 3.4 Pharmacological modulators of Piezo1

Through high-throughput screening assays, specific agonists of Piezo1, such as Yoda1 and Jedi1/2, have been identified (88, 89). Jedi1/2 exhibit superior water solubility compared to Yoda1. Co-administration of Jedi1 and Yoda1 synergistically potentiate the Piezo1 poking currents, suggesting distinct mechanisms underlying their activation of Piezo1. However, currently known inhibitors of Piezo1 lack specificity, including ruthenium red (RR), gadolinium (Gd^3+^), and the peptide toxin GsMTx-4 (90). Given the elucidation of the molecular structure of Piezo1, we anticipate the emergence of additional ligand candidates targeting Piezo1 in the near future.

## 4 Potential roles of endothelial PIEZO1 in COVID-19

**Figure 2:**
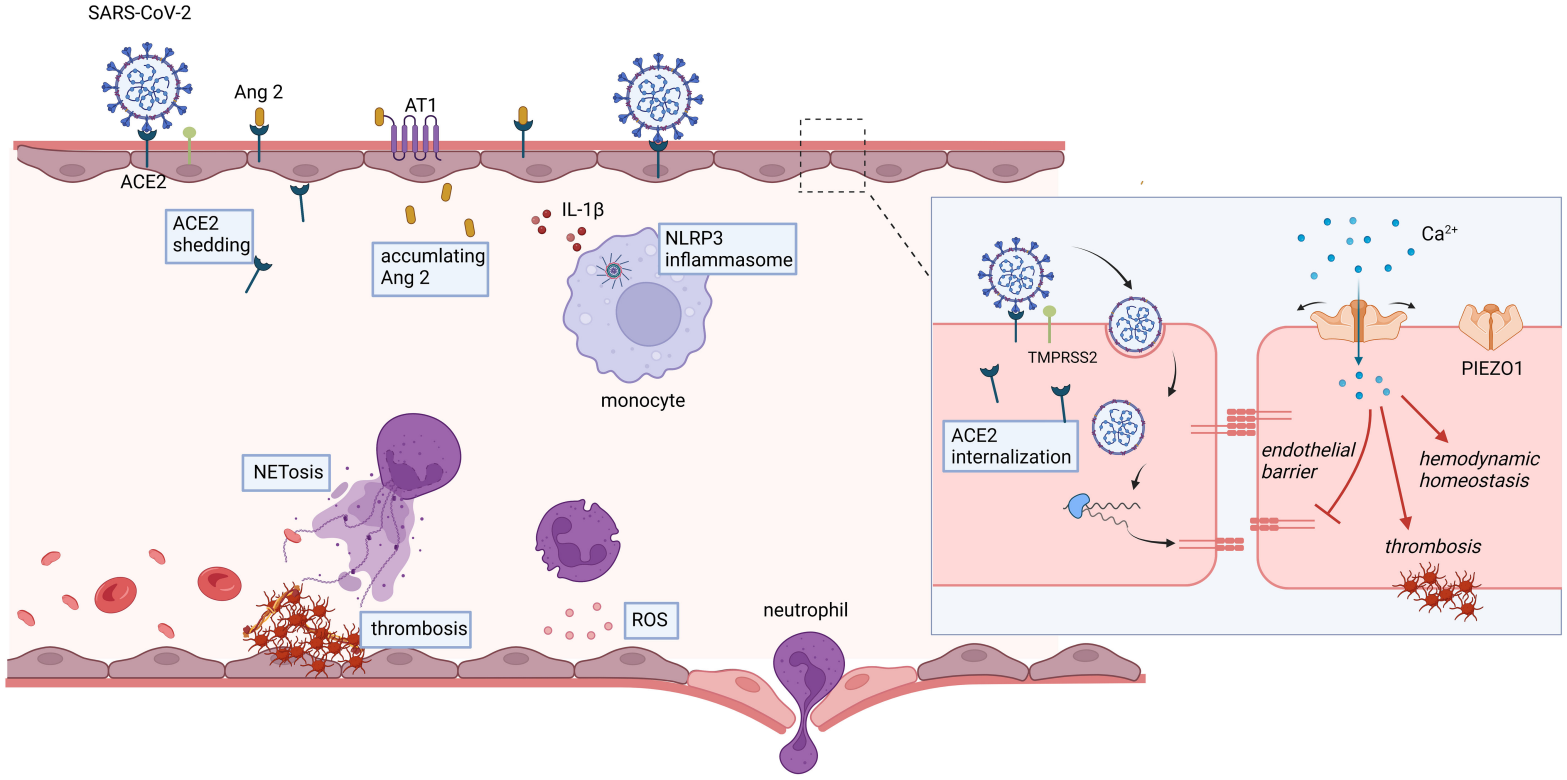
Potential roles of endothelial PIEZO1 in COVID-19.

Endothelial cells serve as a critical interface between the circulatory system and surrounding tissues, playing a multitude of functions in physiological homeostasis and pathological conditions. These roles include regulating vascular permeability, modulating inflammatory responses, maintaining coagulation balance, and promoting angiogenesis (6, 91). The ability of endothelial cells to sense and respond to mechanical stimuli is crucial for maintaining vascular homeostasis. It is well-established that MS channels play a pivotal role in converting mechanical forces into electrochemical signals. Specifically, MS channels in endothelial cells are involved in various events such as vasodilation, vascular inflammation, and vascular permeability. Endothelial PIEZO1 holds promising potential in the treatment of COVID-19, particularly in maintaining systemic hemodynamic stability, endothelial barrier integrity, and muscle capillary density; however, it may also contribute to a procoagulant tendency under certain circumstances (**Figure 2**).

### 4.1 Potential links between PIEZO1 and SARS-CoV-2 induced calcium disturbance

SARS-CoV-2 has been elucidated to interact with calcium channels, potentially leading to profound physiological perturbations (92, 93). The spike protein of SARS-CoV-2 features a critical region known as the spike protein receptor-binding domain (S-RBD), which exhibits a high affinity for human ACE-2 receptors and is crucial for viral entry (94, 95). S-RBD has been implicated in inducing an acute to prolonged increase in intracellular calcium concentration in human pulmonary arterial endothelial cells, which is associated with the activation and expression of Piezo1 and store-operated calcium channels (SOCC) (96). The SARS-CoV-2 induced persistent perturbation of intracellular calcium homeostasis, driven by the upregulation of Piezo1 and other calcium channels, may lead to elevated apoptosis and impairment of pulmonary vascular endothelial cells, contributing to the vascular complications observed in severe COVID-19 cases.

Additionally, the S-RBD disrupts intracellular calcium homeostasis and triggers cell apoptosis by engaging with the ACE2 receptor and formation of ACE2-bounded calcium channel clusters (96). The formation of clusters involving ACE2, Piezo1, and SOCC, induced by the S-RBD, suggests a complex interplay between the virus and the host’s calcium signaling system. These clusters enable the opening of Piezo1 and SOCC, and their formation is dependent on the S-RBD-ACE2 interaction and the presence of intracellular calcium. The use of KobA, which blocks the interaction between ACE2 and S-RBD, has been shown to significantly reduce the S-RBD-induced upregulation and activation of Piezo1, as well as baseline calcium levels. This indicates that the S-RBD’s interaction with ACE2 disrupts intracellular calcium homeostasis, leading to cell apoptosis and the formation of calcium channel clusters, which are critical for the virus’s pathogenic effects.

Collectively, the potential link between Piezo1 and SARS-CoV-2 induced calcium disturbance highlights the intricate relationship between viral mechanisms and host cellular responses. Although there is currently no direct evidence supporting Piezo1 as a receptor for SARS-CoV-2 infection, it is worth considering Piezo1 as a promising target to modulate calcium disturbance in COVID-19 associated endothelial dysfunction.

### 4.2 Potential role of PIEZO1 in maintaining hemodynamic homeostasis

The endothelium is consistently exposed to shear stress induced by blood flow and cyclic pressure, which are essential for sustaining hemodynamic stability. As a Ca^2+^-permeable non-selective cation channel, Piezo1 functions as a vital sensor of blood flow. Patapoutian et al. demonstrated that Piezos mediate neuronal sensing of blood pressure and the baroreceptor reflex (97). Additionally, Piezo1 serves as a key regulator of flow-induced ATP release and shear stress-induced NO production (98). Mice with inducible, endothelium-specific Piezo1 deficiency exhibited reduced endothelial nitric oxide synthase (eNOS) activity, resulting in impaired NO synthesis and the development of arterial hypertension. NO, as an essential vascular vasodilator, also possesses potent anti-inflammatory, anti-apoptotic, and anti-thrombotic properties. Clinical reports have indicated the potential benefits of inhaled NO in the treatment of COVID-19 (99, 100). Collectively, these studies suggest that PIEZO1 could represent a prospective target for regulating and preserving normal hemodynamics in COVID-19 by maintaining hemodynamic homeostasis.

### 4.3 Potential role of PIEZO1 in the maintenance of endothelial barrier

An autopsy study published in The New England Journal of Medicine in 2020 revealed three distinct pulmonary vascular features in patients with COVID-19, *i.e.*, severe endothelial injury, widespread vascular thrombosis, and augmented intussusceptive angiogenesis in the lungs (101). Given its high expression in the lungs, Piezo1 might play an essential role in regulating inflammation and maintaining pulmonary endothelial integrity. A recent study demonstrated that endothelial Piezo1 promotes angiogenesis and facilitates bone fracture repair (102), suggesting its potential involvement in angiogenesis and vascular remodeling during early SARS-CoV-2 infection in the lungs.

Extravasation of leukocytes is a pivotal hallmark of the inflammatory response. A previous study has demonstrated that during the process of leukocyte transendothelial migration, blood flow shear stress intervenes, leading to an increase in membrane tension in vascular endothelial cells. This mechanical force is detected by Piezo1 ion channels, subsequently initiating intracellular calcium elevation and activating endothelial cells. Consequently, endothelial cell contraction ensues, resulting in barrier opening and ultimately facilitating leukocyte transmigration. Thus, Piezo1 functions as a critical mediator whereby vascular endothelial cells orchestrate leukocyte transmigration and contribute to the progression of vascular inflammation (103).

A recent preclinical study provides compelling evidence for the first time that a single exposure to the spike protein or receptor-binding domain of SARS-CoV-2 is sufficient to induce acute-to-prolonged damage to pulmonary vascular endothelium. This damage occurs through the upregulation and activation of Piezo1 and store-operated calcium channels, leading to increased intracellular calcium concentrations. Pharmacological inhibition of Piezo1 using GsMTx4 effectively prevents disruption of intracellular calcium homeostasis in endothelial cells. These findings strongly suggest that targeting Piezo1 could be a potential therapeutic strategy for mitigating S-RBD-induced pulmonary vascular damage, offering novel insights into the management of COVID-associated pulmonary vascular diseases and long-term complications in individuals with long COVID (96). It has been also reported to enhance lung endothelial barrier function and mitigate ventilator-induced lung injury by suppressing Src-induced VE-cadherin phosphorylation (7). Additionally, Piezo1-mediated mechanosensation is critical for innate immunity (5), as infiltrating monocytes recognize cyclic hydrostatic pressure in the lungs through Piezo1, triggering neutrophil-mediated bacterial clearance in mice. These findings collectively suggest that targeting PIEZO1 could represent a promising therapeutic approach for mitigating pulmonary vascular damage in COVID-19.

### 4.4 Potential role of PIEZO1 in the regulation of thrombosis

Piezo1 dysregulation has recently been identified in patients with type 2 diabetes mellitus (T2DM) through perturbational screening (9). Notably, increased Piezo1 activity in platelets, red blood cells, and neutrophils in T2DM leads to a prothrombotic cellular response. Inhibiting Piezo1 may offer a potential strategy for mitigating thrombosis, particularly in the context of hyperglycemia. Another study revealed that activated Piezo1 in platelets of hypertensive mice resulted in significant platelet activation, whereas Piezo1 inhibition improved mitochondrial dysfunction and platelet apoptosis (104). Patients with severe COVID-19 exhibit elevated markers of platelet apoptosis, including heightened depolarization of mitochondrial inner transmembrane potential, cytosolic Ca^2+^ concentration, and phosphatidylserine externalization (105). Targeting procoagulant platelets and platelet apoptosis may hold therapeutic significance for the treatment of COVID-19. Overall, PIEZO1 exhibits important potential in regulating COVID-19-associated thrombosis.

### 4.5 Potential role of PIEZO1 in the regulation of ventilator-induced lung injury

Ventilator-induced lung injury (VILI) is a critical concern in the field of intensive care, referring to the damage or worsening of lung function caused by mechanical ventilation. Mechanical ventilation is commonly required for patients with acute hypoxemic respiratory failure caused by SARS-CoV-2 infection. In severe cases of COVID-19 pneumonia, prolonged use of positive airway pressure (PAP) for more than 24 hours can lead to potential complications, including lung injury due to excessive tidal volume (106). Several mechanisms contribute to VILI, namely barotrauma, volutrauma, atelectrauma, and biotrauma (107). These mechanisms result in alveolar distention and injury, leading to increased alveolar permeability, edema in the alveoli and interstitium, alveolar hemorrhage, and the formation of hyaline membranes. These pathological changes collectively impair surfactant function and ultimately lead to alveolar collapse (108).

Mechanical ventilation applies cyclic and intermittent expansion and distension to the lungs, which in turn exerts mechanical forces on different lung cells via the mechanosensitive ion channel known as Piezo1. These stretching forces are also transmitted to endothelial cells, leading to increased tension on the underlying plasma membrane. Recent studies have primarily aimed to comprehend the involvement of Piezo1 in the progression of VILI. A study conducted by Malik et al. in 2019 found that long-term mechanical ventilation (38 h, 108 h, and 252 h) led to significantly reduced expression of Piezo1 in lung tissue compared to short-term ventilation (less than 1 h) (7). However, in a rat model, increased Piezo1 expression was observed in lung tissue following high-volume ventilation and cyclic stretch treatment. Suppression of Piezo1 activity using GSMTx4 alleviated VILI in rats, resulting in decreased edema, diminished protein leakage, attenuated systemic inflammation, and improved survival rates (109). In another rat model of acute respiratory distress syndrome (ARDS), high tidal volume ventilation during ARDS activated the Piezo1 channel and downstream calpain. Excessive mechanical stretch further exacerbated lung vascular hyperpermeability in ARDS rats, but pharmacological inhibition of calpain or Piezo1 knockdown prevented the disassembly of endothelial adherens junctions and improved endothelial barrier function (110).

Mechanical stretching during ventilation also affects pulmonary epithelial cells. In a murine model involving mechanical ventilation following acid aspiration-induced lung injury, Piezo1 played a pivotal role by mediating calcium influx and adenosine triphosphate (ATP) release in epithelial cells. Targeting epithelial Piezo1 may be a potential therapeutic strategy for preventing pulmonary fibrosis in ARDS patients undergoing mechanical ventilation (111). Furthermore, stretch-induced Piezo1 activation triggers the proteolytic activity of metalloproteinases ADAM10 and ADAM17 at the plasma membrane of primary human lung epithelial cells, resulting in the shedding of the epithelial junctional adhesion molecule-A (JAM-A) and affecting epithelial permeability (112).

Although there is limited research on MS ion channels in the context of COVID-19-associated endothelial dysfunction, a recent preclinical investigation has shown promising results regarding the pharmacological inhibition of Piezo1 using GsMTx4 (96). This inhibition helps maintain intracellular calcium homeostasis in endothelial cells and may hold potential for managing pulmonary vascular disorders associated with COVID-19 and long-term complications in individuals with long COVID. In short, mechanical ventilation can activate PIEZO1 in various lung cells, contributing to lung injury. It should be mentioned that these studies primarily focus on models with mechanical ventilation, highlighting the need for further investigations into lung injuries caused by COVID-19 or other diseases.

### 4.6 Potential role of PIEZO1 in the regulation of sarcopenia

Severe sarcopenia has been observed in certain patients with COVID-19. Contributing factors include immobilization, mechanical ventilation, systemic inflammation, malnutrition, and the targeting of ACE2 by SARS-CoV-2 in skeletal muscle (113, 114). As previously discussed, endothelial Piezo1 serves as a sensor for blood flow and activates eNOS to facilitate nitric oxide (NO) synthesis. Remarkably, NO suppresses thrombospondin-2, an inducer of endothelial cell apoptosis, which is predominantly expressed in muscle pericytes. Endothelial Piezo1 supports muscle capillary density and sustains physical activity through the endothelial cell-pericyte interaction within the muscle microenvironment (115). A recent study demonstrated that immobilization leads to reduced cytosolic calcium concentration in skeletal muscle cells, potentially due to Piezo1 downregulation. Acute disruption of Piezo1 promotes skeletal muscle atrophy involving KLF15 and IL-6 pathways (116). These findings indicate that activating endothelial PIEZO1 represents a promising therapeutic approach for COVID-19-associated sarcopenia.

### 4.7 Potential role of PIEZO1 in the regulation of long COVID

Long COVID is characterized by prolonged symptoms following COVID-19 and has become a significant global healthcare concern (117). The spectrum of symptoms observed in individuals with long COVID encompasses fatigue, brain fog, respiratory complications, and cardiovascular abnormalities (118). It is now widely recognized that the persistence of these symptoms is not solely attributable to viral persistence, but rather involves a complex interplay of factors including viral reservoir, systemic inflammation, endothelial dysfunction, and organ damage (119).

Endothelial dysfunction is one of the key features of long COVID and has been linked to thrombotic events and impaired oxygen delivery. The presence of microclots have been observed in both acute COVID-19 and long COVID (120, 121). Long-term alterations in the size and stiffness of blood cells have also been identified in individuals with long COVID, potentially leading to impaired oxygen delivery (122). In addition, patients with long COVID exhibited a persistent decrease in vascular density, particularly in small capillaries (123). Therefore, endotheliitis and coagulation have been recognized as two important targets for the multifaceted therapeutic approach to long COVID treatment (124, 125). Given the potential involvement of PIEZO1 in regulating vascular homeostasis, inflammation, endothelial barrier integrity, and thrombotic processes, targeting PIEZO1 may hold therapeutic promise for alleviating or preventing the symptoms associated with long COVID.

The endothelial PIEZO1 demonstrates significant therapeutic potential in the context of COVID-19 therapeutics, due to its crucial role in preserving systemic hemodynamic stability and ensuring the integrity of the endothelial barrier. Nevertheless, it may also elicit a procoagulant propensity under certain conditions.

## 5 Challenges in utilizing PIEZO1 as a therapeutic target for COVID-19

The role of PIEZO1 in endothelial dysfunction following SARS-CoV-2 infection is still not well understood, but its involvement in other inflammatory disorders suggests its potential as a target for clinical research. However, there is currently limited clinical evidence supporting PIEZO1 as a therapeutic target for COVID-19. In a study conducted in 2020 by Sukumar et al., the analysis of data from the UK Biobank revealed a significant association between COVID-19 fatality and three missense SNPs within the PIEZO1 gene, independent of established risk factors (11). It is worth noting that these SNPs affect amino acid residues in the unexplored proximal N-terminus region of the PIEZO1 protein. Furthermore, variations in the prevalence of these SNPs across different ethnic groups were observed through a comprehensive analysis of genomic sequences. These findings provide some evidence of PIEZO1’s potential contribution to COVID-19 fatality and its association with ethnic susceptibility.

Despite the promise of PIEZO1 as a therapeutic target for COVID-19, clinical research on its potential remains limited compared to basic scientific studies. Before considering its clinical application, several significant challenges need to be addressed. Firstly, the precise role of PIEZO1 in the pathogenesis of COVID-19 remains unclear, as the mechanisms underlying SARS-CoV-2-induced inflammation, thrombosis, and other complications are complex and multifaceted. Understanding the specific contributions of PIEZO1 in these processes is essential for targeted interventions. Additionally, careful examination of the off-target effects of PIEZO1 modulation is necessary. PIEZO1 is ubiquitously expressed in various tissues and cell types throughout the body, playing crucial roles in diverse physiological functions. Modulating PIEZO1 may inadvertently disrupt normal cellular processes, leading to unintended consequences. Developing safe and effective therapeutics that selectively target PIEZO1 poses another challenge. Small molecule inhibitors, gene silencing techniques, and other strategies must undergo rigorous testing to determine their specificity, bioavailability, and potential side effects.

## 6 Conclusion and prospective

In light of the ongoing presence and long-term impact of the COVID-19, it is crucial to prioritize reducing mortality and mitigating post-COVID syndrome. Recent evidence suggests that endothelial injury induced by SARS-CoV-2 infection plays a significant role in organ dysfunction. Therefore, it is imperative to comprehend the implications of COVID-19-associated endothelial dysfunction for the development of effective therapeutic strategies. Although investigations into PIEZO1 as a potential therapeutic target for COVID-19 remain limited, extensive research has elucidated the multifaceted biological functions of the PIEZO1 ion channel in vascular physiology. Notably, studies have shown a correlation between genetic variants of PIEZO1 and COVID-19 fatality. Preclinical investigations have also demonstrated that exposure to the S-RBD of SARS-CoV-2 can impair pulmonary vascular endothelium, activating Piezo1. Pharmacological inhibition of Piezo1 effectively prevents disruption of endothelial calcium homeostasis. Furthermore, basic research has revealed that regulating PIEZO1 can ameliorate endothelial dysfunction and improve VILI in animal models. These findings highlight the therapeutic potential of targeting PIEZO1 in mitigating COVID-19-associated endothelial dysfunction.

Future research should focus on unraveling the precise molecular mechanisms underlying PIEZO1-mediated endothelial dysfunction and identifying specific signaling pathways activated by PIEZO1. Additionally, exploring the safety and efficacy of PIEZO1 modulators through clinical trials will be essential for translating these findings into clinical practice. Further studies are warranted to validate endothelial PIEZO1 as a novel target for developing therapeutics against COVID-19. Additionally, investigating the contributions of other mechanosensitive calcium channels in COVID-19-associated endothelial dysfunction may provide further insights into the complex mechanotransduction pathways involved.

In summary, understanding the role of mechanosensitive ion channels, particularly PIEZO1, in COVID-19-associated endothelial dysfunction holds great promise for the development of novel therapeutic interventions aimed at restoring endothelial homeostasis and improving patient outcomes. Leveraging rational drug design and innovative endothelial-targeted nanoparticles, targeting PIEZO1 may offer a promising approach to promote vascular protection in the context of COVID-19.

## Author contributions

XZ: Conceptualization, Writing – original draft. JL: Writing – review & editing. XD: Conceptualization, Supervision, Writing – review & editing. LB: Conceptualization, Supervision, Writing – review & editing.
